# Classification of power quality disturbances in microgrids using a multi-level global convolutional neural network and SDTransformer approach

**DOI:** 10.1371/journal.pone.0317050

**Published:** 2025-02-12

**Authors:** Junzhuo Jiang, Hao Wu, Changhua Zhong, Hong Song

**Affiliations:** 1 Automation and Information Engineering, Sichuan University of Science & Engineering, Yibin, Sichuan, China; 2 Artificial Intelligence Key Laboratory of Sichuan Province, Yibin, Sichuan, China; 3 Aba Teachers University, Aba, Sichuan, China; Huaiyin Institute of Technology HYIT: Huaiyin Institute of Technology, CHINA

## Abstract

As the adoption of new energy sources like photovoltaic and wind power increases alongside the influx of advanced power electronic devices, there has been a significant rise in power quality disturbance events (PQDs) within power systems. These disturbances, including harmonics and voltage dips, severely impact the stability of microgrids and the efficiency of power equipment. To enhance the accuracy of identifying power quality disturbances in microgrids, this paper introduces a Multi-level Global Convolutional Neural Network combined with a Simplified double-layer Transformer model (MGCNN-SDTransformer). The model processes the input raw 1D time-series signals of power quality through multi-level convolutional and 1D-Global Attention Mechanism (1D-GAM) operations in MGCNN, which preliminarily extracts and emphasizes the key features and dynamic changes; Subsequently, the model utilizes the Multi-head Self Attention(MSA) and Multi-Layer Perceptron(MLP) components of the enhanced SDTransformer to further explore the transient local and periodic global features of the signals; The classification outcomes are then determined using a fully-connected layer and a Softmax classifier. The model effectively retains the signal’s original one-dimensional temporal attributes while also delving into more complex features. This approach exhibits strong resistance to noise and enhanced generalization skills, markedly improving the detection accuracy of power quality issues within microgrids.

## Introduction

Microgrids, compact and standalone power systems, encompass diverse distributed power sources, loads, storage options, and control mechanisms. They provide crucial benefits including efficient use of resources, swift deployment, and broad versatility, making them excellent solutions for energy management challenges. The extensive deployment of power electronics within microgrids introduces substantial harmonic signals to the electrical grid, potentially degrading power quality through issues like voltage waveform distortions, fluctuations, flicker, and imbalances across phases. Microgrids operate as autonomous nodes, functional in both interconnected and standalone settings, which optimizes the use of distributed resources. This setup not only supports local energy management and enhances grid robustness but also minimizes energy loss during transmission. By synchronizing local energy production with consumption demands, microgrids effectively alleviate peak load stresses and stabilize voltage levels, thereby boosting the reliability of the supply and increasing the efficiency of the grid. As critical components of advanced power system research, microgrids help tackle major operational and stability challenges in power systems. One of the key technologies in microgrids involves the precise detection of power quality disturbances(PQDs), including voltage sags, harmonic distortions, and interruptions, which is vital for future power quality management strategies [1].

Global scholars are currently honing in on the precise identification of PQDs signals. Research [2] has pioneered the use of a wavelet transform approach that integrates empirical wavelet transform, Hilbert transform, and singularity decomposition for pinpointing harmonic component characteristics. Research [3] has presented a novel framework that combines the Recurrence Plot (RP) method with ResNet-50 CNN for classifying power quality disturbances. RP transforms 1-D signals into 2-D images, from which ResNet-50 extracts features for classification using a support vector machine. Additionally, Research [4] has proposed a PQD detection method combining parameter-optimized variational mode decomposition (VMD) with improved wavelet thresholding, using the Improved Dung Beetle Optimizer (IDBO) for faster and more accurate decomposition, achieving superior noise reduction, higher localization accuracy, and improved signal-to-noise ratio (SNR). Literature [5] employs real-time data generation, preprocessing, feature extraction, and selection, alongside intelligent modeling using an SVM classifier that utilizes feature vectors of the intrinsic mode function (IMF) generated by EMD. This approach outperforms the traditional LVQ-NN method.

With the continuous development of the smart grid, power data are also increasing rapidly, and traditional shallow machine learning techniques struggle to extract useful information from this massive data set. Among these techniques, converting one-dimensional signals into two-dimensional images has gained popularity among scholars. Literature [6] proposes a method to diagnose power quality disturbances using a continuous wavelet transform (CWT) and a convolutional neural network (CNN). The CWT converts disturbances into two-dimensional spectrogram images normalized to 128x128 pixels, enabling the CNN to efficiently identify six types of disturbances. Literature [7] proposed a PQD classification method that utilizes the Gramian Angular Field (GAF) to convert one-dimensional signal data into two-dimensional images. This method completes the PQD classification using transfer learning with an enhanced AlexNet model. Literature [8] uses the Markov Transition Field (MTF) to convert one-dimensional power quality disturbance time series signals into two-dimensional visual maps. These maps are then processed using deep residual networks to extract features and accurately classify the disturbances.

Many scholars have frequently reused the temporal model that accounts for the one-dimensional characteristics of PQD signals and avoids complex signal upscaling operations. Literature [9] assessed the effectiveness of various temporal model architectures, including CNN, RNN, I-RNN, LSTM, GRU, and CNN-LSTM, through experiments that optimized these architectures with specific parameters and topologies for synthetic and real-time power quality disturbance analysis. Literature [10] developed a hybrid model combining CNN and Bidirectional Long Short-Term Memory (Bi-LSTM) using an inverse signaling approach, effectively demonstrating its capabilities through experimentation. Literature [11] employs a CNN combined with Long Short-Term Memory (LSTM) to automatically detect and classify features in PQD signals.

Although the existing deep learning-based PQDs recognition has achieved good results, the image model still needs to upgrade the original PQDs signal, and the RNN and its derived temporal models are not effective in dealing with the long-term dependence of the signal.The steady-state perturbations such as harmonics and voltage gaps in PQDs signals are periodic, and such global features tend to be extracted at low depths in both the RNN and CNN, so that deeper features cannot be extracted for recognition by using only CNN. The Transformer model was proposed to solve the text translation problem. Unlike CNNs that use convolutional operations, the Transformer uses an attentional mechanism to capture global features in the input that have long-term dependencies, avoiding the inherent drawbacks of RNNs and CNNs, and has good application prospects, but only using the Transformer model has good results. It has good prospects for application, but the Transformer model cannot be used for processing one-dimensional signals using only the Transformer model.

To solve the above problems, the contributions of this paper are summarized as follows: A novel one-dimensional global attention mechanism (1D-GAM) has been developed to refine existing models by incorporating channel and sequence attention mechanisms, streamlining the processing of time-series signals and significantly enhancing the attention layer’s focus for precise identification and augmentation of key features and dynamics in the data. Complementing this, a multi-layer global convolutional neural network (MGCNN) features variably scaled convolutional kernels in each layer to detect both minor and major signal disturbances, efficiently processing one-dimensional signals through multiple convolution and activation layers to extract complex features and enrich temporal and spatial information for sophisticated analysis. Additionally, a redesigned SDTransformer model is proposed for one-dimensional power quality signal processing, eliminating positional encoding and integrating a Multilayer Perceptron (MLP) with a dropout layer to shift the model’s focus to signal features over sequence positions, enhancing time-series analysis efficiency. This design improves nonlinear processing capabilities for accurate disturbance pattern recognition while reducing overfitting and improving generalization. Finally, the MGCNN-SDTransformer recognition model combines the spatial and temporal feature extraction strengths of MGCNN with the Multi-head Self-Attention (MSA) mechanism of the SDTransformer, enabling parallel processing across multiple subspaces. This integration significantly enhances the capture of feature interdependencies, offering superior processing efficiency and accuracy by leveraging the advantages of its constituent technologies.

## Mathematical modeling of PQDs

**Fig 1:**
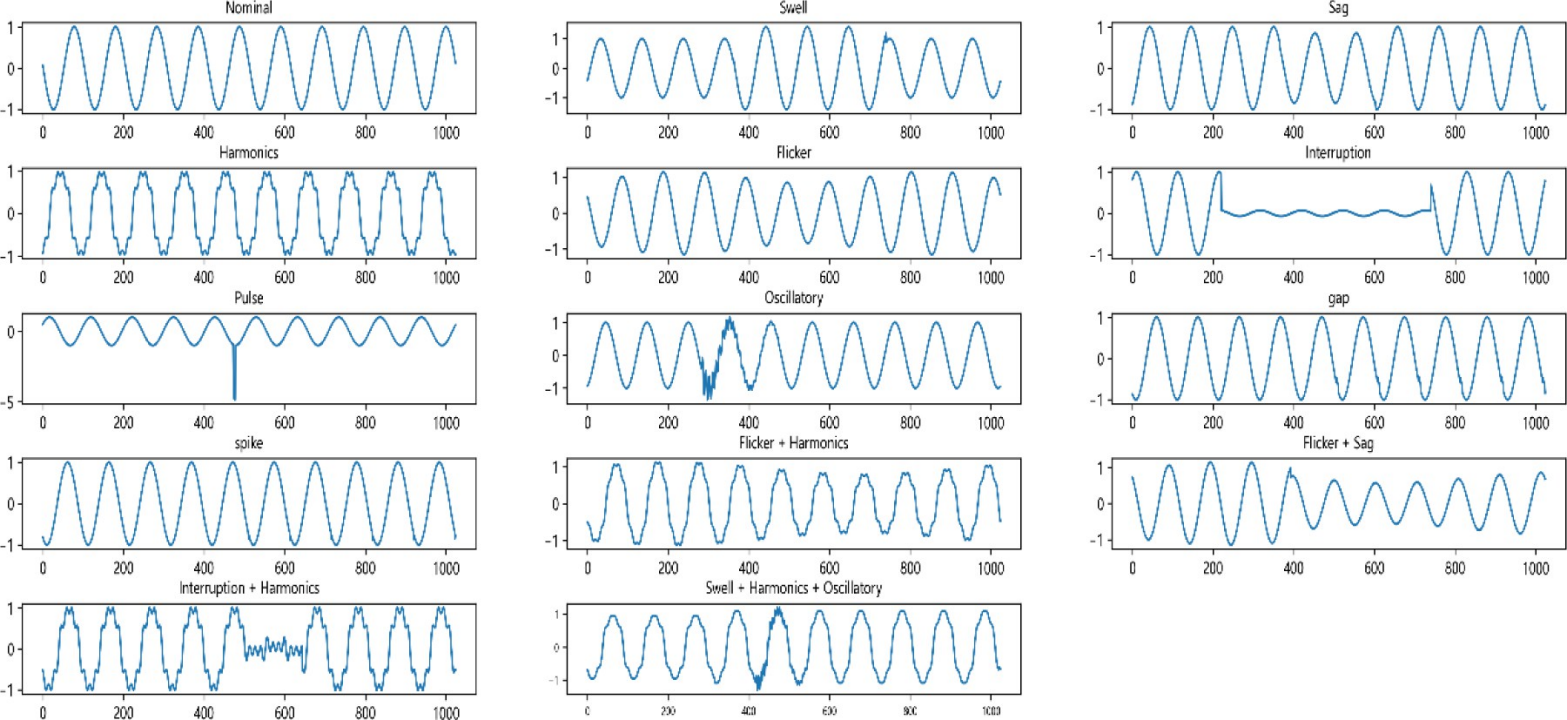
Model of double-circuit transmission line on the same tower.

According to standards such as IEEE 1159–2019 [12], a mathematical model of power quality perturbations in microgrids has been developed, and the common single perturbations in power quality perturbations in microgrids are generally categorized into eight types, i.e., voltage swell, voltage sag, voltage harmonics, voltage flicker, voltage interruption, pulse, voltage oscillatory, voltage gap, and voltage spike. The composite power quality disturbance signal can be considered as the result of the joint action of multiple disturbances of the standard signal. The disturbances act on the standard signal in two ways, multiplication and addition, plus the normal signal, there are nine single disturbances and part of the composite disturbances, as shown in Table 1, ε(t) for the unit step signal. Fig 1 gives some of these disturbance waveforms.

**Table 1:** 9 single disturbance and some composite disturbances.

Disturbance type	Mathematical model	Restrictive condition
Nominal	$y(t)=\left[1\pm \alpha \left(u\left(t-t_{1}\right)-u\left(t-t_{2}\right)\right)\right]\sin\;(\omega t)$	$\alpha <0.04,\;T\leq t_{2}-t_{1}\leq 9T$
Swell	$y(t)=\left[1+\alpha \left(u\left(t-t_{1}\right)-u\left(t-t_{2}\right)\right)\right]\sin\;(\omega t)$	$0.1\leq \alpha \leq 0.8,\;T\leq t_{2}-t_{1}\leq 9T$
Sag	$y(t)=\left[1-\alpha \left(u\left(t-t_{1}\right)-u\left(t-t_{2}\right)\right)\right]\sin\;(\omega t)$	$0.1\leq \alpha \leq 0.9,\;T\leq t_{2}-t_{1}\leq 9T$
Harmonics	$y(t)=\alpha_{1}\;\sin\;(\omega t)+\alpha_{3}\;\sin(3\omega t)+\alpha_{5}\;\sin(5\omega t)$ $+\alpha_{7}\sin\;(7\omega t)$	$0.05\leq \alpha_{3},\alpha_{5},\alpha_{7}\leq 0.15,\Sigma \left(\alpha_{i}^{2}\right)=1$
Flicker	$y(t)=\left[1+\alpha_{f}\sin\;(\beta \omega t)\right]\sin\;(\omega t)$	$0.1\leq \alpha_{f}\leq 0.2,5\leq \beta \leq 20$
Interruption	$y(t)=\left[1-\alpha \left(u\left(t-t_{1}\right)-u\left(t-t_{2}\right)\right)\right]\sin\;(\omega t)$	$0.9\leq \alpha \leq 1,\;T\leq t_{2}-t_{1}\leq 9T$
Pulse	$y(t)=\left[1-\alpha \left(u\left(t-t_{1}\right)-u\left(t-t_{2}\right)\right)\right]\sin\;(\omega t)$	$0.1\leq \alpha \leq 0.414,0.05T\leq t_{2}-t_{1}\leq 0.1T$
Oscillatory	$\begin{array}{l} y(t)=\sin\;(\omega t)+\alpha^{-\left(t-t_{1}\right)/\tau }\sin\;\left(\omega_{n}\left(t-t_{1}\right)\right)\times \\ \left(u\left(t-t_{2}\right)-u\left(t-t_{1}\right)\right) \end{array}$	$0.1\leq \alpha \leq 0.8,0.5T\leq t_{2}-t_{1}\leq 3T,$ $\tau \leq 40,300\leq f_{n}\leq 900$
Gap	$\begin{array}{l} y(t)=\sin\;(\omega t)-sign\;(\sin\;(\omega t))\times \\ \sum_{i=1}^{n}k\left[u\left(t-\left(t_{1}-0.02n\right)\right)-u\left(t-\left(t_{2}-0.02n\right)\right)\right] \end{array}$	$0\leq t_{1},t_{2}\leq 0.5T,0.1\leq \;K\leq 0.4,$ $0.01T\leq t_{2}-t_{1}\leq 0.05T$
Spike	$\begin{array}{l} y(t)=\sin\;(\omega t)+sign\;(\sin\;(\omega t))\times \\ \sum_{i=1}^{n}\;k\left[u\left(t-\left(t_{1}-0.02n\right)\right)-u\left(t-\left(t_{2}-0.02n\right)\right)\right] \end{array}$	$0\leq t_{1},t_{2}\leq 0.5T,0.1\leq \;K\leq 0.4,$ $0.01T\leq t_{2}-t_{1}\leq 0.05T$
Flicker + Harmonics	$\begin{array}{l} y(t)=\left[\alpha_{1}\sin\;(\omega t)+\alpha_{3}\sin\;(3\omega t)+\alpha_{5}\sin\;(5\omega t)+\right.\\ \left.\alpha_{7}\sin\;(7\omega t)\right]\times \left[1+\alpha_{f}\sin\;(\beta \omega t)\right] \end{array}$	$0.1\leq \alpha_{f}\leq 0.2,5\;Hz\leq \beta \leq 20\;Hz,$ $0.05\leq \alpha_{3},\alpha_{5},\alpha_{7}\leq 0.15,\Sigma \left(\alpha_{i}^{2}\right)=1$
Flicker + Sag	$y(t)=\left[1+\alpha_{f}\sin\;(\beta \omega t)\right]\times \left[1-\alpha \left(u\left(t-t_{1}\right)-u\left(t-t_{2}\right)\right)\right]\sin\;(\omega t)$	$0.1\leq \alpha_{f}\leq 0.9,\;T\leq t_{2}-t_{1}\leq 9\;T$ 0.1≤α≤0.2,5Hz≤β≤20Hz
Interruption + Harmonics	$\begin{array}{l} y(t)=\left[1-\alpha \left(u\left(t-t_{1}\right)-u\left(t-t_{2}\right)\right)\right]\times \\ \left[\alpha_{1}\sin\;(\omega t)+\alpha_{3}\sin\;(3\omega t)+\alpha_{5}\sin\;(5\omega t)+\alpha_{7}\sin\;(7\omega t)\right] \end{array}$	$0.9\leq \alpha \leq 1,\;T\leq \left(t_{2}-t_{1}\right)\leq 9T$ $0.05\leq \alpha_{3},\alpha_{5},\alpha_{7}\leq 0.15,\Sigma \left(\alpha_{i}^{2}\right)=1$
Swell + Harmonics + Oscillatory	$y(t)=\left[1+\alpha \left(u\left(t-t_{1}\right)-u\left(t-t_{2}\right)\right)\right]+$ $\left[\alpha^{-\left(t-t_{1}\right)/\tau }\sin\;\left(\omega_{n}\left(t-t_{1}\right)\right)\times \left(u\left(t-t_{2}\right)-u\left(t-t_{1}\right)\right)\right]\times$ $\left[\alpha_{1}\sin\;(\omega t)+\alpha_{3}\sin\;(3\omega t)+\alpha_{5}\sin\;(5\omega t)+\alpha_{7}\sin\;(7\omega t)\right]$	$0.1\leq \alpha \leq 0.8,\;T\leq t_{2}-t_{1}\leq 9T,$ $\tau \leq 40,300\leq f_{n}\leq 900,$ $0.05\leq \alpha_{3},\alpha_{5},\alpha_{7}\leq 0.15,\Sigma \left(\alpha_{i}^{2}\right)=1$

### Multi-level global convolutional neural network and SDTransformer recognition model

Different from traditional methods, the multi-level global convolutional neural network and SDTransformer recognition model based on multi-level global convolutional neural network and SDTransformer proposed in this paper takes the original PQDs one-dimensional signals as the input without any complex preprocessing operations, integrates the feature extraction with the disturbance recognition, and directly outputs the classification results.

### Multi-level global convolutional neural network

#### 1. One-dimensional global attention mechanism

**Fig 2:**
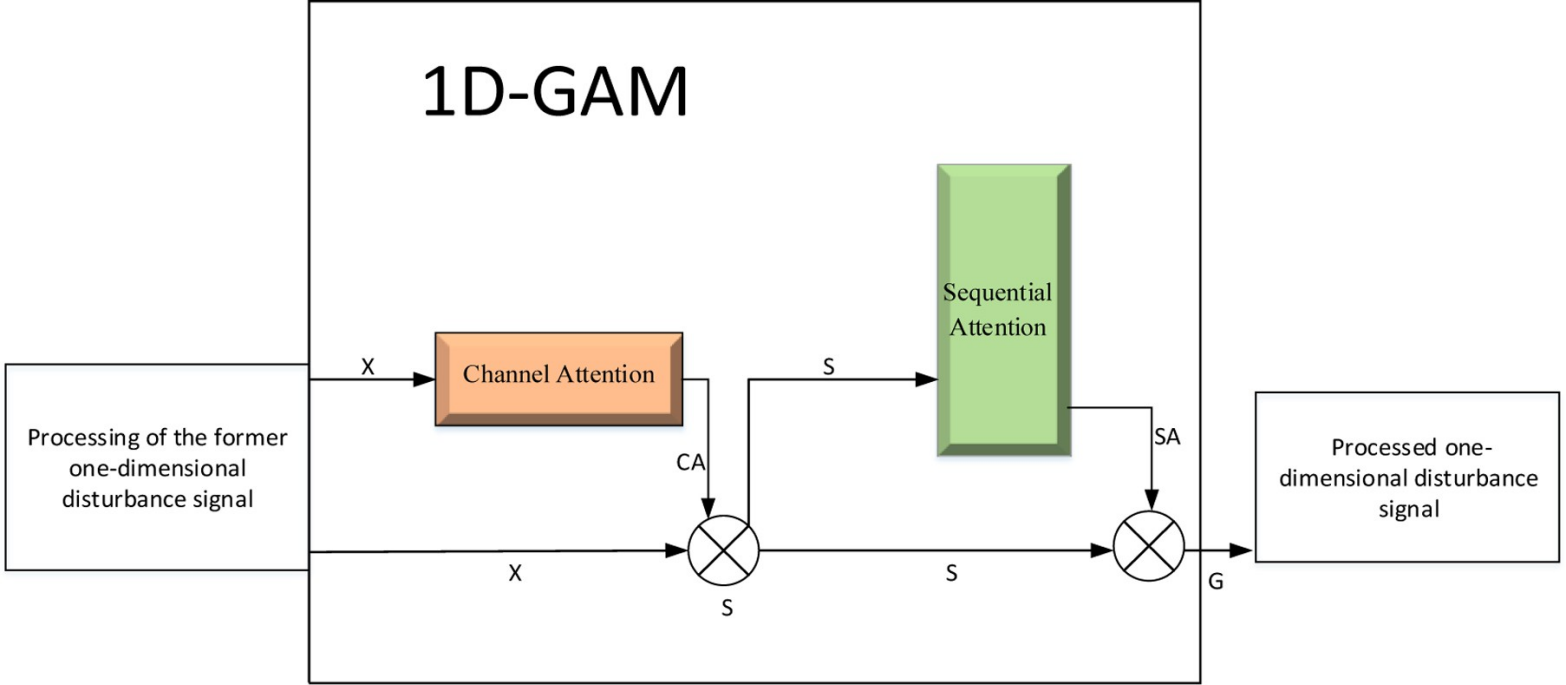
One-dimensional global attention mechanism (1D-GAM) module.

In this paper, we enhance the Global Attention Mechanism (GAM) [13] by introducing a 1D-GAM that is optimized for signal processing. This mechanism comprises two main components: a channel attention sub-module and a sequence attention sub-module designed specifically for 1D data. The architecture utilizes a hybrid attention approach that effectively focuses on both channel and sequence dimensions, which is essential for capturing complex dependencies in 1D signals. This is illustrated in Fig 2.

*1*.*1 The channel attention mechanism*. The Channel Attention mechanism operates via a simple feed-forward network comprising two linear transformations. This mechanism dynamically recalibrates the channels of the input feature signals. Below, we detail the specific network structure and computational process:

Let the input feature signal be X∈R^b×c×l^, where b is the batch size, c is the number of channels (notably, c = 1 for one-dimensional signals), and l is the sequence length. The calculation of channel attention proceeds as follows:

1. Channel Compression: Initially, the channel dimension of the input feature signal X is reduced using a fully connected layer—a linear transformation—with $\mathrm{int}\left(\frac{c}{rate}\right)$ output units, where "rate" is a hyperparameter defining the compression ratio. This step aims to diminish the model’s complexity and parameter count while preserving essential information in Eq (1).

$$Z_{1}=W_{1}X+b_{1}$$ (1)

where $W_{1}\in R^{\mathrm{int}\left(\frac{c}{rate}\right)\times c}$ and $b_{1}\in R^{\mathrm{int}\left(\frac{c}{rate}\right)}$ are the weights and biases of that linear layer.

2. Nonlinear Activation: Subsequently, the ReLU activation function [14] is applied to introduce nonlinearity, enhancing the model’s ability to capture complex feature dependencies as detailed in Eq (2).

A=ReLUZ1=max0,Z1 (2)

3. Channel Recovery: Finally, another linear layer restores the channel dimension from $\mathrm{int}\left(\frac{c}{\;rate}\right)$ to the original c to complete the computation of channel attention. This process enhances significant channel features while diminishing the less relevant ones in Eq (3).

$$Z_{2}=W_{2}\;A+b_{2}$$ (3)

where $W_{2}\in R^{c\times \mathrm{int}\left(\frac{c}{\;rate}\right)}$ and b_2_∈R^c^ are the weights and biases of that linear layer.

4. Output Scaling: The dimensions of the output channel attention feature signal remain consistent with the input. The final output is scaled using a sigmoid function [15], which restricts values to the range [0,1], thereby quantifying the importance of each channel in Eq (4).

$$CA=\sigma \left(Z_{3}\right)=\frac{1}{1+e^{-Z_{3}}}$$ (4)

where σ() denotes the sigmoid function.

5. Importance Weighting: Following this processing, the channel attention mechanism outputs the importance weights for each channel. These weights dynamically adjust the feature responses of each channel by applying the Hadamard product [16] with the original input, effectively scaling each channel’s features within the input signal in Eq (5).

S=X⊙CA (5)

where ⊙ denotes the Hadamard Product, which S is the adjusted feature signal that reinforces important feature channels and suppresses others, which helps in the subsequent model learning and decision making process.

*1*.*2 Sequential attention*. The Sequential Attention mechanism, specifically designed for one-dimensional data, enhances important features and suppresses unimportant ones within the sequence dimension of the feature map through the sequential processing of two one-dimensional convolutional layers. The specific implementation of this mechanism includes the following steps:

Let the output channel attention feature signal S dimension be R^b×c×l^, where b is the batch size, c is the number of channels, and l is the sequence length. The computation of sequence attention can be divided into the following steps:

1. First Convolutional Layer: This layer, employing a convolutional kernel of size 7 with a stride of 1 and padding of 3, aims to reduce the number of channels through convolutional operations without altering the sequence length. The number of output channels, $\mathrm{int}\left(\frac{c}{\;rate}\right)$, depends on the rate, a hyperparameter controlling channel compression in Eq (6).

$$Y_{1}=H_{1}*\;S+b_{3}$$ (6)

where $H_{1}\in R^{\mathrm{int}\left(\frac{c}{\;rate}\right)\times c\times 7}$ and $b_{3}\in R^{\mathrm{int}\left(\frac{c}{\;rate\;}\right)}$ are the weights and * bias of the convolution kernel indicating the convolution operation.

2. Batch Normalization and ReLU Activation: the convolved feature signals are processed through the Batch Normalization layer (BN) [17] and ReLU activation function to stabilize the training process and introduce nonlinearities, thereby enhancing the model’s ability to capture complex patterns in Eq (7).

Y2=ReLUBNY1 (7)

where BN denotes the Batch Normalization operation.

3. Second Convolutional Layer: The subsequent convolutional layer, using a kernel size of 7 and padding of 3, increases the number of channels from $\mathrm{int}\left(\frac{c}{rate}\right)$ back to the final number of output channels out_c_hannels , maintaining constant sequence length in Eq (8).

$$Y_{3}=H_{2}*Y_{2}+b_{4}$$ (8)

where $H_{2}\in R^{out_{C}hannels\times \mathrm{int}\left(\frac{c}{rate}\right)\times 7}$ and $b_{4}\in R^{out_{c}\;hannels}$ are the weights and bias of the convolution kernel.

4. Output Batch Normalization: Finally, the processed signal Y_3_ is normalized using another batch normalization layer, yielding the final sequence attention feature signal, denoted as Y_4_ in Eq (9).

$$Y_{4}=BN\left(Y_{3}\right)$$ (9)

5. Applying Sigmoid Function: The sequence attention feature signal Y_4_ undergoes processing via the Sigmoid activation function, which assigns an importance weight for each position, with values ranging from [0, 1] in Eq (10).

$$SA=\sigma \left(Y_{4}\right)=\frac{1}{1+e^{-Y_{4}}}$$ (10)

where σ() denotes the Sigmoid function.

6. Integration with Input: With this design, the output of the sequence attention module SA, specifically the sequence attention feature signal, is element-wise multiplied (Hadamard product) with the input. This operation dynamically adjusts feature responses based on the calculated importance weights in Eq (11).

G=S⊙SA (11)

which ⊙ denotes the Hadamard Product,G is the feature signal adjusted by sequence attention. This mechanism effectively strengthens the feature response at important locations in the sequence, improves the model’s ability to capture key information, and helps to improve the overall performance of the model.

#### 2. Multi-level global convolutional neural network

**Fig 3:**
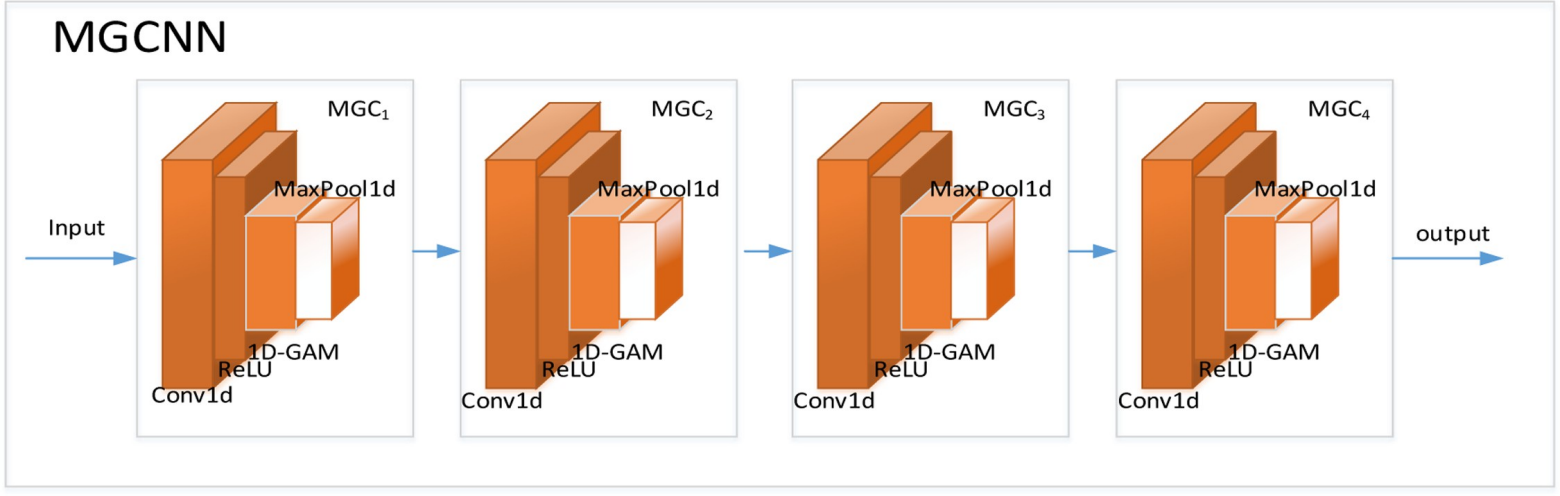
MGCNN structure.

The MGCNN advances the traditional one-dimensional CNN by incorporating multiple convolution layers and a global attention mechanism. This architecture integrates complex convolution operations with attentive processing, significantly boosting the network’s performance. The MGCNN uses convolutional blocks in four distinct layers to process one-dimensional power quality disturbances with varied convolution depths. These layers work together to refine input data at multiple scales, improving the network’s detection and characterization capabilities. Activation functions are employed to enhance the network’s nonlinear modeling, effectively extracting features across different channels and sequences from the signals. The structure of the MGCNN is depicted in Fig 3.

The mathematical model of the Multi-level Global Convolution (MGC) layer is detailed in Eq (12) below:

$$MGC_{i}=F_{pool}\;\left(F_{sa}\;\left(F_{ca}\;\left(F_{relu}\;\left(F_{conv}\;(X)\right)\right)\right)\right)\;i=1,2,3,4$$ (12)

Where F_conv_ () represents a 1D convolutional layer, F_relu_ () is a ReLU activation layer, F_ca_ () denotes a channel attention layer, F_sa_ () signifies a sequence attention layer, and F_pool_ () is a maximum pooling layer.

The model processes a one-dimensional input signal with a length of 1024 and a single channel. The model structure consists of several components, each with the following functions:

One-dimensional convolutional layer (Conv1d): This layer employs a one-dimensional convolutional kernel to extract features from the sequential signal. It transforms the single-channel input into a multi-dimensional feature representation. The operation of this layer is mathematically represented in this paper by the following equation in Eq (13):

$$F_{conv}\;(X)=W_{i}*X+b_{i}$$ (13)

where F_conv_ (X) is the output feature signal, W_i_ and b_i_ are the weights and bias of the ith convolutional layer, respectively, and *X* is the input feature signal, * representing the convolution operation.

GAM Attention Module: This module comprises two parts: channel attention and sequence attention. The channel attention component models the dependencies between channels using linear transformations, whereas the sequence attention component concentrates on emphasizing important features within the sequence.

Maximum Pooling Layer (MaxPool1d): This layer downsamples the feature signals to reduce their size and expand the receptive field, thereby enhancing the model’s ability to capture relevant features at various scales. The specific parameters are shown in Table 2 below.

**Table 2:** MGCNN model specific parameters.

Hierarchical numbering	Hierarchical type	Patch Size/Stride	Output Size	Depth
	Input	64x1x1024		1
MGC_1_	Conv1d	64x16x1024	3/1	16
	ReLU	64x16x1024		16
	1D-GAM(Channel Attention)	64x16x1024		16
	1D-GAM(Sequential Attention)	64x16x1024	7/1	16
	MaxPool1d	64x16x512	2/2	16
MGC_2_	Conv1d	64x32x512	3/1	32
	ReLU	64x32x512		32
	1D-GAM(Channel Attention)	64x32x512		32
	1D-GAM(Sequential Attention)	64x32x512	7/1	32
	MaxPool1d	64x32x256	2/2	32
MGC_3_	Conv1d	64x64x256	3/1	64
	ReLU	64x64x256		64
	1D-GAM(Channel Attention)	64x64x256		64
	1D-GAM(Sequential Attention)	64x64x256	7/1	64
	MaxPool1d	64x64x128	2/2	64
MGC_4_	Conv1d	64x128x128	3/1	128
	ReLU	64x128x128		128
	1D-GAM(Channel Attention)	64x128x128		128
	1D-GAM(Sequential Attention)	64x128x128	7/1	128
	MaxPool1d	64x128x64	2/2	128

After the one-dimensional power quality disturbance signals have been processed by the MGCNN model into deeper feature signals, these signals are then inputted into the SDTransformer to further extract and enhance the features.

#### 3. The SDTransformer model

Since its introduction by Vaswani et al. in 2017, the Transformer model [18] has revolutionized the field of Natural Language Processing (NLP). In our research, we adapt this seminal architecture to enhance the processing and parsing capabilities for time series data, aiming to leverage its robust feature extraction for more complex sequential patterns.

In traditional applications, the Transformer model requires positional encoding to process sequence data effectively, as it lacks inherent capabilities to recognize the positional relationships within the sequence. However, in architectures like our model’s MGCNN component that incorporate CNN, it is feasible to omit positional encoding. This adaptation simplifies the structural design and reduces computational complexity while preserving performance efficacy.

**Fig 4:**
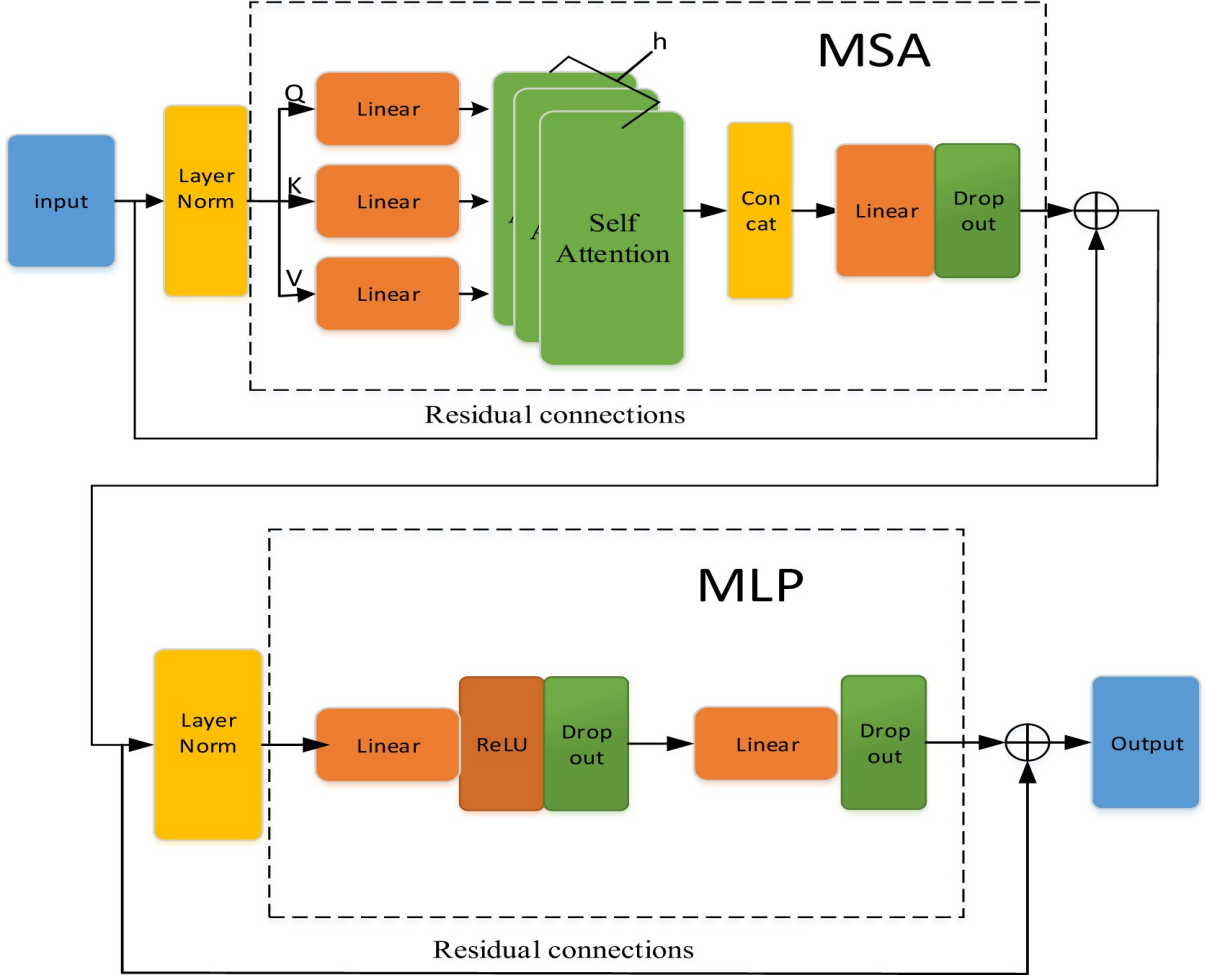
SDTransformer base model.

In this paper, the MLP module with residual structure is introduced to improve the original Transformer model, and the original Transformer model is simplified into only two-layer base structure to obtain the SDTransformer (Simplified double-layer SDTransformer) model. An SDTransformer base module consists of one MSA module, one MLP module, two residual modules and two normalization modules, as shown in Fig 4.

1. Multi-head Self Attention (MSA) [19]: This module is central to the SDTransformer architecture; it distributes the self-attention mechanism across multiple heads, allowing the model to simultaneously process information in different subspaces. This parallel processing capability enables it to detect complex patterns and relationships within the data. Each head independently processes specific aspects of the input data and then combines them into a unified output representation. For a given query (Q), key (K) and value (V), the MSA is calculated as shown in Eq (14):

$$MultiHead\;(Q,\;K,\;V)=Concat\;\left(head_{1},\ldots ,\;head_{h}\right)W^{O}$$ (14)

Each head’s computation is detailed separately in Eq (15):

$$head_{i}=Attention\left(QW_{i}^{Q},KW_{i}^{K},VW_{i}^{V}\right)=\mathrm{softmax}\;\left(\frac{\left(QW_{i}^{Q}\right){\left(KW_{i}^{K}\right)}^{T}}{\sqrt{\;d_{k}}}\right)VW_{i}^{V}$$ (15)

$W_{i}^{Q}$, $W_{i}^{K}$, $W_{i}^{V}$, is the weight matrix of the query, key, and value corresponding to that header. W^O^ is the weight matrix of all header outputs that are spliced and then linearly transformed. d_k_ is the dimension of the key vector, which is used to scale the dot product to prevent the internal dot product from being too large and affecting the gradient of softmax.

2. Multi-layer Perceptron (MLP) [20]:To improve the SDTransformer’s nonlinear feature extraction capabilities, a MLP module is utilized. This module typically includes two linear layers separated by a ReLU activation function. It processes the output from the MSA unit, applying nonlinear transformations that enhance the model’s expressive power, as outlined in Eq (16):

MLP(x)=σW2ReLUW1x+b1+b2 (16)

W_1_,W_2_ is the weight matrix of the linear layer, b_1_,b_2_ is the bias term.ReLU is a nonlinear activation function, which is usually used to increase the nonlinear processing power.σ denotes the nonlinear transformation after the activation function.

The model incorporates Dropout layers in critical components, including the MSA and MLP effectively minimizing the risk of overfitting.

3. Residual connections [21]: The outputs of each sub-module (MSA and MLP) in the SDTransformer model are added back to the inputs through residual connections. This structural design helps to avoid the problem of gradient vanishing that may occur in the deep network during the training process, Eq (17):

LayerOutput=LayerInput+Sublayer(LayerInput) (17)

where Sublayer can be an MSA or MLP module. By adding the inputs directly to the outputs, the residual connections enable the deep network to learn constant mappings, thus maintaining network performance while preventing gradient problems.

4. Layer Norm [22]: The introduction of the normalization module before both the MSA and MLP layers is followed by a normalization layer (usually layer normalization). The normalization layer normalizes the inputs to have a mean of 0 and a variance of 1, which contributes to the stability of the model training and the speed of convergence in Eq (18).

$$LayerNorm\;(x)=\gamma \left(\frac{x-\mu }{\sigma }\right)+\beta$$ (18)

μ is the mean of the data to be normalized. σ is the standard deviation of the data to be normalized.γ,β is the learnable parameter used to recover the original distribution of the normalized data.

#### 4. MGCNN-SDTransformer-based PQDs recognition framework

**Fig 5:**
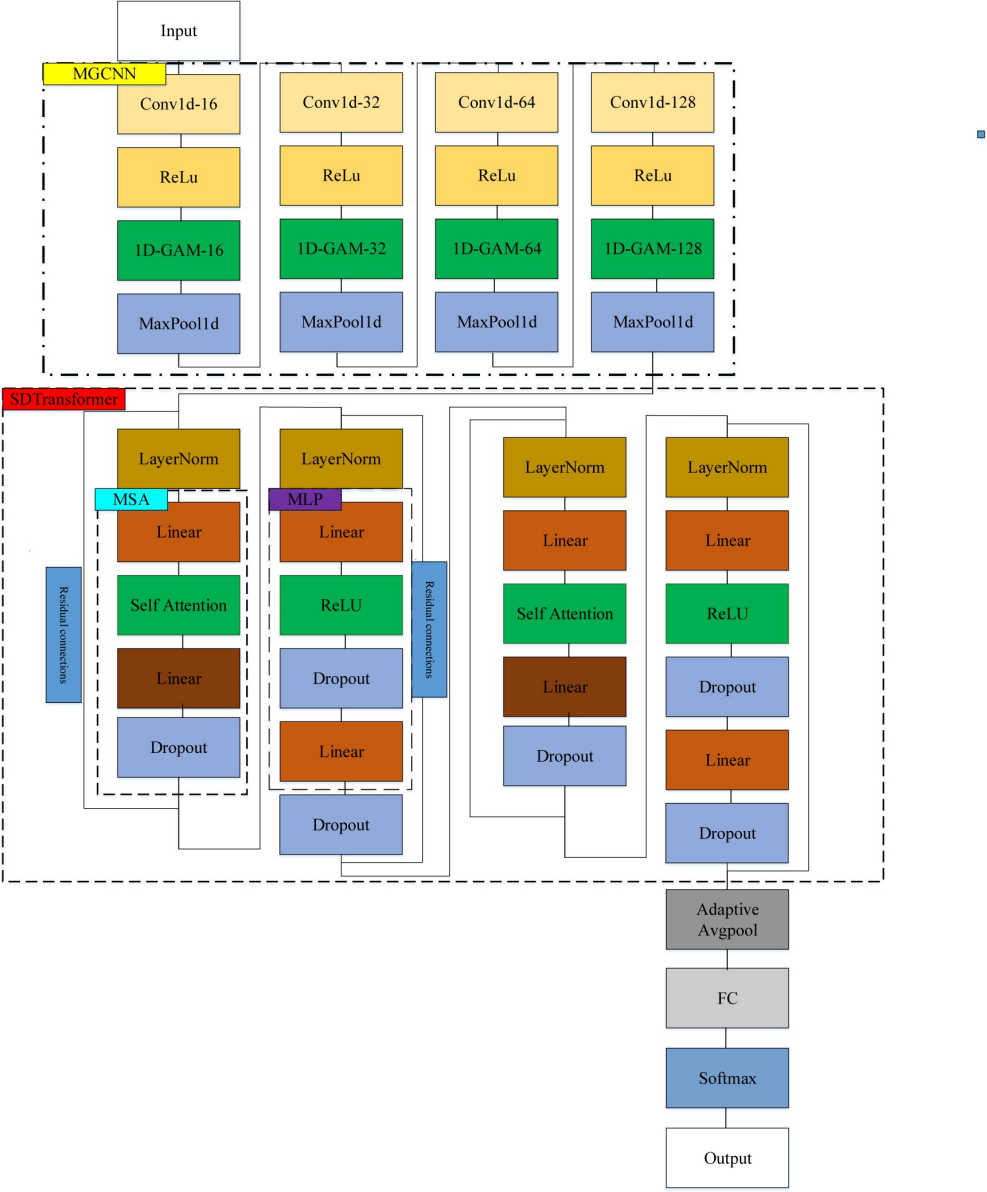
PQDs recognition framework based on MGCNN-SDTransformer.

The PQDs recognition framework using the MGCNN-SDTransformer model is depicted in Fig 5, combining feature extraction and disturbance recognition into a single workflow as follows:

1. The model processes one-dimensional power quality disturbance signals, typically containing various noises like transient interruptions. These signals first undergo MGCNN, where each layer, enhanced by the 1D-GAM, captures and integrates features across both channel and sequence dimensions. This multilayer approach not only reveals fine-grained details at lower levels but also synthesizes them into a comprehensive signal representation at higher levels.
2. The SDTransformer model processes extracted features using a self-attention mechanism to highlight significant attributes and suppress less relevant ones. It employs MSA to analyze signals across various subspaces simultaneously, which improves the detail and accuracy of feature interdependency representation. Additionally, the model incorporates a MLP for advanced nonlinear feature extraction and employs Dropout layers and residual connections to stabilize training and prevent issues like gradient vanishing. This comprehensive strategy not only enhances the model’s accuracy in detecting various types of power quality disturbances but also boosts computational efficiency, facilitating fast and precise classification in complex scenarios. An adaptive pooling layer standardizes feature dimensions, ensuring consistent output sizes regardless of the input’s original scale.
3. The SDTransformer-optimized features are processed through a fully connected layer and a Softmax classifier for final classification. This layer consolidates the outputs into a lower-dimensional space, enhancing disturbance identification effectiveness, facilitated by the Softmax classifier.

## Simulation and analysis

### Generation of PQD dataset

Under the IEEE 1159–2019 standard, we establish mathematical models for 29 types of power quality disturbances, categorized into 10 single, 15 double, and 4 triple disturbances. These are uniquely numbered and utilize a solo thermal coding scheme, as detailed in Table 4. The normal signal is counted as a special disturbance in training the model, totaling 29 power quality disturbance signals. The fundamental frequency is set to 50Hz, the sampling frequency is set to 5.12kHz, and the sampling duration is 10 fundamental cycles, totaling 1024 sampling points. We generated 29 PQDs randomly in environments with noise levels of noise-free, 50 dB, 30 dB, and 20 dB, using randomized amplitudes and occurrence times. These signals were then combined to create a mixed-noise dataset. Each disturbance type comprises 1200 samples, split into training, validation, and test sets in a 7:1:2 ratio. The training, validation, and test sets are distinct with no overlap; the training set trains the model, whereas the validation and test sets evaluate its generalization performance.

### Model parameters and training results

The MGCNN-SDTransformer model proposed in this paper is built based on the VSCode deep learning framework and python, and the training environment parameters are shown in Table 3.

**Table 3:** Training environment parameters.

Hardware/Software	Model/Version
Operating System	Windows(64-bit)
CPU	Intel Core i5-12600Kf 3.7GHz
GPU	GeForce RTX 3060Ti
Python	3.10.9
VSCode	1.87.2

**Fig 6:**
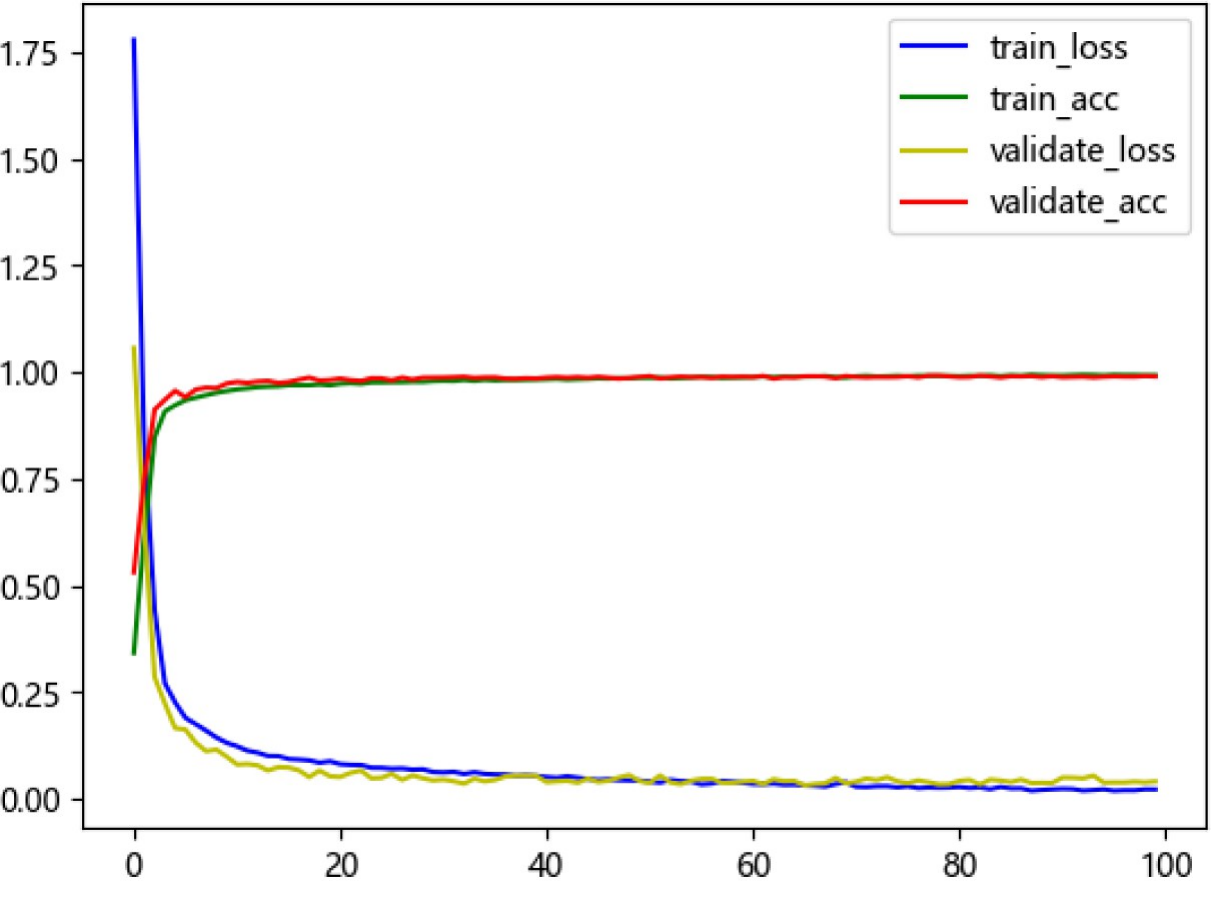
Model training process.

The MGCNN network comprises 4 module layers, the SDTransformer base modules have a depth of 2, and the MSA modules feature 4 attention heads each. The loss function for training is the cross-entropy function [23], commonly used in classification problems, and is expressed as follows:

$$Loss=-\frac{1}{\;B}\sum_{p=1}^{B}\sum_{q=1}^{Q}y_{pq}\log\;\left({\hat{y}}_{pq}\right)$$ (19)

where is the training batch size, is the number of PQDs categories, and are the true label and predicted probability of the first input in the first category of PQDs, respectively. The recognition Accuracy and Loss value for the training and test sets during model training are shown in Fig 6.

Fig 6 shows that the model achieves over 95% accuracy within 20 iterations, maintaining this high level with rapid convergence and without overfitting. The recognition accuracies of various types of perturbations under different noise levels are shown in Table 4.

**Table 4:** 29 Categories of recognition accuracy.

Labels	Disturbance type	Noise-free(%)	50dB(%)	30dB(%)	20dB(%)
C0	Nominal	100	100	98.65	91.18
C1	Swell	100	98.59	100	95
C2	Sag	100	100	100	100
C3	Harmonics	100	100	100	100
C4	Flicker	100	95	97.75	98.67
C5	Interruption	98.84	98.82	98.72	100
C6	Pulse	100	100	100	98.89
C7	Oscillatory	100	100	100	100
C8	gap	100	97.78	100	100
C9	spike	100	100	100	100
C10	Harmonics+Swell	100	100	100	98.57
C11	Harmonics+Sag	100	100	100	98.77
C12	Harmonics+Interruption	100	100	98.89	98.65
C13	Harmonics+Flicker	100	100	98.82	100
C14	Harmonics+Pulse	100	100	100	100
C15	Harmonics+Oscillatory	100	100	100	100
C16	Flicker+Swell	100	100	100	100
C17	Flicker+Sag	100	100	96.05	98.81
C18	Flicker+Oscillatory	100	100	100	100
C19	Flicker+Pulse	100	100	100	100
C20	Flicker+Interruption	97.47	97.53	95.38	93.26
C21	Swell+Oscillatory	100	100	100	100
C22	Sag+Oscillatory	100	100	100	100
C23	Swell+Pulse	100	100	100	100
C24	Sag+Pulse	100	100	100	100
C25	Harmonics+Oscillatory+Swell	98.67	100	100	98.61
C26	Harmonics+Oscillatory+Sag	98.86	100	100	100
C27	Harmonics+Oscillatory+Interruption	95.71	96.2	98.73	97.3
C28	Harmonics+Oscillatory+Flicker	100	100	100	100
Total		99.64	99.55	99.41	98.85

Table 4 exhibits the detection accuracy of various types of power quality disturbances (e.g., normal signal, voltage transient rise, voltage transient drop, etc.) under different noise levels (Noise-free, 50db, 30db, and 20db). Overall, the model is robust across various noise environments; although accuracy decreases slightly from 99.64% in noise-free conditions to 98.85% at 20 dB, it remains impressively high.

Notably, categories such as C2 (voltage transients), C3 (voltage harmonics), and C7 (voltage oscillations) consistently achieve 100% accuracy across all noise levels, underscoring the model’s effectiveness in detecting these disturbances. This emphasizes the efficiency and accuracy of the model in identifying these power quality disturbances. However, performance drops in categories like C20 (flicker + interruptions) are notable, where accuracy falls to 93.26% at 20 dB. This drop may point to the limitations of the model in dealing with composite power quality disturbances (i.e., cases containing multiple anomalous features) in the context of high noise.

Despite its overall excellent performance and robustness, the system can still be improved to handle specific or composite disturbances better, especially in high-noise settings.

### Comparison experiment of different preprocessing

To evaluate the impact of different preprocessing methods on power quality disturbance signals, we compared four methods—raw data, empirical modal decomposition (EMD) [24], wavelet packet transform (WPD) [25], and discrete wavelet transform (DWT) [26]—keeping model parameters constant throughout the experiments. The results indicate how each preprocessing method affects recognition accuracy under various noise levels (Noise-free, 50 dB, 30 dB, and 20 dB).

Table 5 demonstrates the advantages and performance of each preprocessing method in enhancing model recognition accuracy, but the performance of the methods diverges as the noise level increases. EMD has relatively low accuracy in all noise conditions, WPD and DWT show high accuracy in all noise environments, especially DWT has high recognition accuracy in all noise conditions, although the difference between the accuracy of DWT and the original data is very small, but the original data do not need to be processed by the complex preprocessing, which can reduce the time and resource consumption of data processing. Therefore, the final choice is to directly input the raw data into the MGCNN-SDTransformer model.

**Table 5:** Comparison of model recognition accuracy.

Different treatments	Noise-free(%)	50dB(%)	30dB(%)	20dB(%)
EMD	98.64	98.41	98.21	97.88
WPD	99.83	98.61	98.74	98.7
DWT	99.65	99.35	99.2	98.71
Raw Data	99.64	99.55	99.41	98.85

### Comparison experiments of different levels of global convolution modules

To examine the impact of various convolution module depths on the MGCNN-SDTransformer model, experiments were conducted with the Multi-level Convolution Block (MC) conFigd at 2, 3, and 4 layers without the 1D-GAM, in a 30 dB noise environment, keeping all other parameters constant. These tests compared accuracy, training time, and the number of parameters.

According to Table 6, the MGC module consistently outperforms the MC module in accuracy across all layer configurations, highlighting the global attention mechanism’s role in enhancing model precision. Specifically, at a 4-layer configuration, the MGC module achieves a 99.41% accuracy rate, significantly surpassing the MC module. In addition, as the number of layers increases, the accuracy of both modules shows an improvement trend, but the speed of improvement slows down with the increase in the number of layers, indicating that increasing the number of layers can effectively improve the model performance, but the effect has a decreasing trend.

**Table 6:** Comparison of different levels of global convolution modules.

Layers of Convolution Module	30dB(%)	Training time (s)	number of parameters
MC = 2	96.92	1139	27997
MC = 3	97.66	826	76669
MC = 4	98.18	806	235453
MC = 5	98.52	836	798781
MGC = 2	97.99	1212	33357
MGC = 3	99.01	987	98733
MGC = 4	99.41	1106	323693
MGC = 5	98.96	1401	1150445

The training time for the MC module does not increase linearly with more layers, suggesting a balance between model complexity and training efficiency. The structural characteristics of the model, the computational complexity, the efficiency of the optimization algorithm, and the data processing flow all have an impact on the training time. The longest training duration, observed in the MGC = 2 configuration, likely results from these combined factors. Conversely, the MGC module’s training time increases with each additional layer, reflecting the global attention mechanism’s higher computational demands. The introduction of the global attention mechanism in the MGC model increases the training time compared to the MC model at all levels, reflecting its higher computational complexity. The MGC module typically requires more parameters than the MC module due to the additional attention weights that need to be learned, which enhances model performance. As the number of layers increases, the number of parameters grows significantly for both modules, consistent with the expectation that more layers lead to more convolutional kernels and connections, thereby increasing model complexity.

In summary, the MGC module, with its global attention mechanism, significantly enhances accuracy over the traditional MC module, though it does require more parameters and longer training times. This highlights the need to balance accuracy, training efficiency, and parameter count in deep learning models, leading to the optimal choice of four layers for the MGC modules.

### Comparison experiments of SDTransformer models with different parameters

In this study, we evaluated the recognition accuracy of PQDs and the model’s training time across various SDTransformer network depths (2, 4, and 6 base layers) and noise levels (Noise-free, 50 dB, 30 dB, and 20 dB), with the multi-head attention mechanism set to 4 heads. The impacts of network depths on recognition accuracy and training time are detailed in Table 7.

**Table 7:** Comparison of different SDTransformer network depths.

Base SDTransformer Layers	Noise-free(%)	50db(%)	30db(%)	20db(%)	Training time (s)
2	99.64	99.55	99.41	98.85	1106
4	99.39	99	98.87	97.87	1285
6	98.91	98.65	98.52	97.35	1394

The data show that all configurations maintain high accuracy in noise-free environments; notably, the 2-layer configuration achieves the highest accuracy at 99.64%, while the 6-layer configuration records the lowest at 98.91%, likely due to overfitting from increased network layers. Given its parameter efficiency and minimal data requirements, the MGCNN-SDTransformer model performs optimally with a shallow configuration of 2 layers.

We fixed the layer number at 2 and maintained other parameters constant to examine how varying MSA head counts (2, 4, 8 heads) affects accuracy and training time across noise levels (Noise-free, 50 dB, 30 dB, 20 dB), as shown in Table 8.

**Table 8:** Comparison of the number of MSA in SDTransformer network.

Number of MSA	Noise-free(%)	50db(%)	30db(%)	20db(%)	Training time (s)
2	99.48	99.09	98.52	98.31	1105
4	99.64	99.55	99.41	98.85	1106
8	99.78	99.2	99.18	98.7	1149

Results indicate that with two fixed encoder layers, increasing the number of attention heads gradually improves recognition accuracy at all noise levels. Specifically, in noise-free conditions, accuracy improves from 99.48% to 99.78% as heads increase from 2 to 8, peaking at 4 heads, which suggests optimal recognition in clearer signal environments. Furthermore, training time mildly rises from 1105 seconds with 2 heads to 1149 seconds with 8 heads. This relatively small increase suggests that increasing the number of heads in the multi-head attention mechanism has a more limited effect on training time. Therefore, in the SDTransformer model, appropriately increasing the number of multi-heads can significantly improve the model’s PQDs recognition accuracy in different noise environments without significantly increasing the training time.

### Ablation experiments between different modules

The results from the ablation experiments, as shown in Table 9, provide insights into the contribution of each module to the overall performance. The CNN model achieves an accuracy of 97.15%, and the MGCNN model shows a slight improvement at 97.65%, indicating the benefit of incorporating multi-scale spatial feature extraction. However, the Transformer model alone performs poorly with an accuracy of 58.16%, highlighting its limitations in power quality disturbance detection without additional modifications. The SDTransformer module is not evaluated independently because it omits positional encoding, which significantly impacts its ability to process sequential data effectively.

**Table 9:** Ablation experiments between different modules.

Module	30db Accuracy (%)
CNN	97.15
MGCNN	97.65
Transformer	58.16
SDTransformer	-
CNN-Transformer	98.05
CNN-SDTransformer	98.18
MGCNN-Transformer	99.1
MGCNN-SDTransformer	99.41

When combining modules, the CNN-Transformer model achieves a notable accuracy of 98.05%, while the CNN-SDTransformer and MGCNN-Transformer models further improve the accuracy to 98.18% and 99.1%, respectively. These results demonstrate that integrating temporal modeling with spatial feature extraction boosts performance. Finally, the MGCNN-SDTransformer combination achieves the highest accuracy of 99.41%, illustrating the synergistic effect of combining MGCNN’s spatial capabilities with the SDTransformer’s temporal processing, resulting in superior performance in detecting power quality disturbances across various noise levels.

### Comparison experiments of different networks

**Fig 7:**
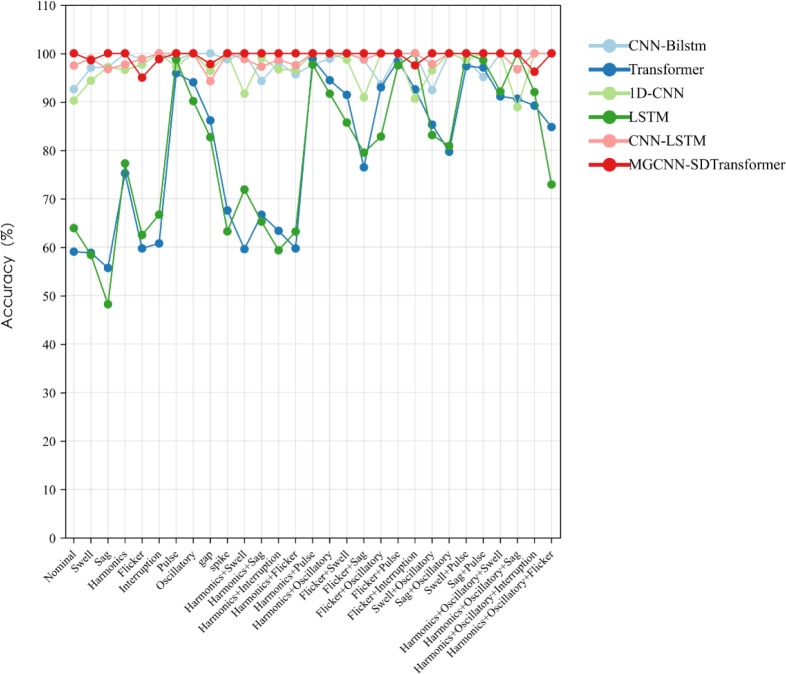
Recognition accuracy of 29 disturbance types under different networks.

**Fig 8:**
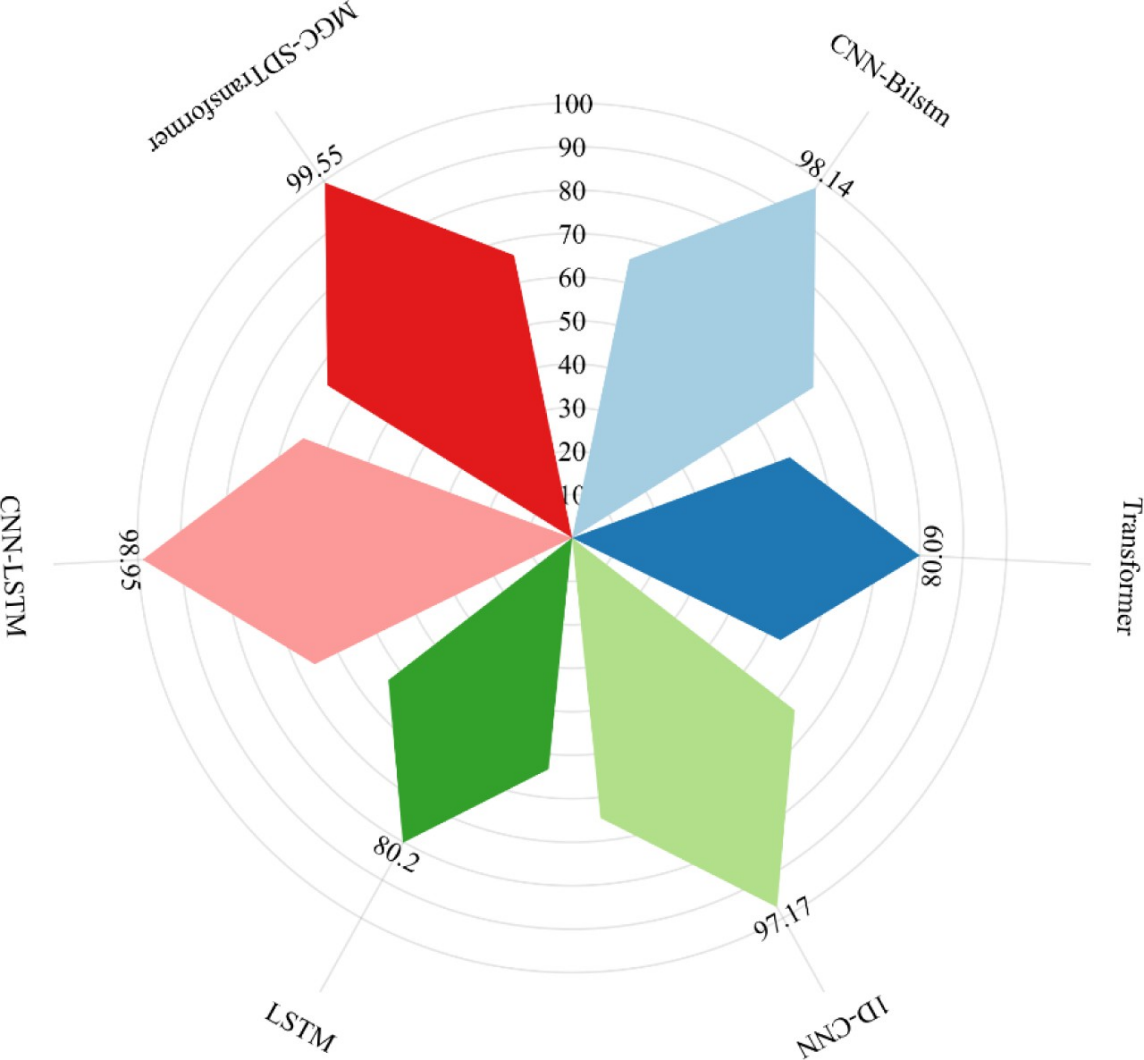
Disturbance integrated recognition rate under different networks.

This study evaluates the impact of different deep learning methods on PQDs classification by comparing five techniques—1D-CNN, CNN-BiLSTM, Transformer, LSTM, and CNN-LSTM—with our MGCNN-SDTransformer method. All models were trained using the same dataset in a 50 dB noise environment over 100 iterations, and their performance was tested by assessing the recognition accuracy and combined recognition rate for 29 types of disturbances, as depicted in Figs 7 and 8.

Figs 7 and 8 show that the CNN-BiLSTM model achieved 98.14% accuracy, demonstrating its capability to capture both forward and backward dependencies in time series data effectively. However, in the PQDs recognition task, the SDTransformer and LSTM models achieved lower accuracies of 80.09% and 80.20%, respectively, despite performing well in other tasks. This suggests that these models may require more specialized architectures to effectively extract relevant features from this type of signal data. Conversely, the 1D-CNN model excels in feature extraction, achieving 97.17% accuracy, particularly with spatially complex data. The CNN-LSTM model, merging CNN’s feature extraction with LSTM’s sequence processing, achieves an accuracy of 98.95%, showcasing the effectiveness of this hybrid architecture. The MGCNN-SDTransformer model proposed in this paper outperforms other models with an accuracy of 99.55%. This high accuracy results from its capability to integrate multidimensional information, including time-domain and frequency-domain features and the long-range dependencies of the time series, which enhances recognition accuracy.

### Comparison experiments with other articles

To validate the MGCNN-SDTransformer model’s performance, we compared it with several state-of-the-art techniques. These methods include both machine learning and deep learning techniques, such as FDST+DT, DWT+PNN, CS+DCNN, GCNN+AFEN, 1D-VGG, DAE, MCF-TST, MTF-EfficientNet and DL-WMV. Each method was evaluated on the PQD identification task at signal-to-noise ratios from 20 dB to 50 dB, detailed in Table 10.

**Table 10:** Comparison of recognition rates of different models.

Methodologies	Number of PQDs	20dB	30dB	Accuracy (%) 40dB	50dB	Noise-free
FDST+DT [27]	13	-	97.49	98.8	99.28	
DWT+PNN [28]	16	93.6	95.2	98.6	-	
CS+ DCNN [29]	15	-	99.81	99.94	-	99.99
GCNN+AFEN [30]	12	96.62	98.98	99.26	-	
1D VGG [31]	17	96.74	98.49	98.93	-	
DAE [32]	15	97.99	98.69	98.93	-	
MCF-TST [33]	29	96.74	99.51	-	99.34	99.33
MTF-EfficientNet [34]	23	96.2	98.12	96.1		99.48
DL-WMV [35]	19	-	98.05	98.58	99.26	-
MGCNN-SDTransformer	29	98.85	99.41	-	99.55	99.64

At 50 dB SNR, the MGCNN-SDTransformer reaches an impressive 99.55% accuracy, which is significantly higher than the other methods, highlighting its superior ability to detect power quality disturbances even in low-noise environments. This result emphasizes the potential of deep learning models, specifically the integration of MGCNN and SDTransformer, in handling complex patterns in noisy data.

Moreover, the MGCNN-SDTransformer maintains high accuracy even in lower SNR conditions. At 20 dB, it achieves 98.85%, and at 30 dB, it reaches 99.41%. These results underscore the robustness of the MGCNN-SDTransformer in real-world scenarios, where noise levels can vary and affect the accuracy of detection. The model’s ability to maintain high performance at these challenging noise levels speaks to its capacity for effective disturbance identification, even in the presence of significant noise.

In contrast, the machine learning-based methods, such as DAE, MCF-TST, and MTF-EfficientNet, show relatively lower accuracy, particularly at lower SNR levels, which indicates their limitations in effectively capturing complex power quality disturbances when noise is present. While these methods can be computationally more efficient, they struggle with robustness in noisy conditions, a crucial aspect for practical applications in power quality monitoring.

Overall, the MGCNN-SDTransformer outperforms all other techniques, demonstrating its robustness, accuracy, and superior performance in detecting power quality disturbances across a wide range of noise conditions. This analysis reinforces the effectiveness of combining MGCNN’s spatial processing with SDTransformer’s temporal modeling, which together provide a powerful solution for PQD identification in real-world environments.

### Real data comparison experiment

**Fig 9:**
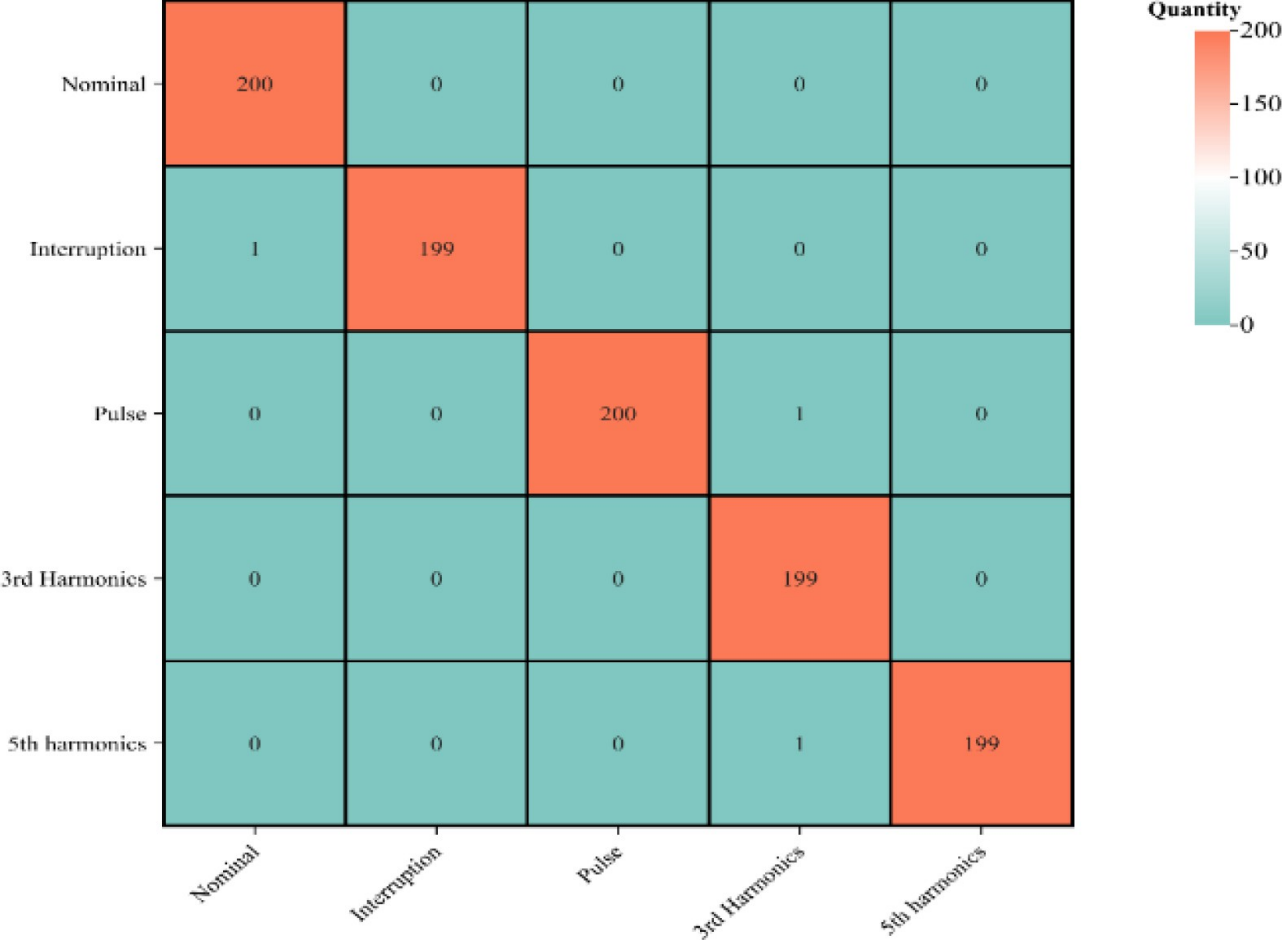
Recognition results of real dataset.

To validate the method’s feasibility, we used a set of real power quality disturbance signals as input for the model discussed in this paper. The dataset, sourced from the Kaggle public database, includes five types of power quality signals: normal signals, voltage interruptions, impulses, third harmonics, and fifth harmonics. Two hundred samples of each signal type were selected for the test set, with results displayed in Table 11 and Fig 9.

**Table 11:** Recognition results of real dataset.

Disturbance type	Sample number	Accuracy(%)
Nominal	200	100
Interruption	200	99.5
Pulse	200	100
3rd Harmonics	200	99.5
5th Harmonics	200	99.5
Average		99.7

Analysis of Table 11 and Fig 9 shows that the model achieves an average recognition accuracy of 99.7%, strongly affirming its capability to identify real power quality disturbances. The model’s high accuracy highlights its reliability and practicality, especially for precise monitoring and diagnosing of power quality issues. However, the dataset primarily comprises single perturbations and lacks a diverse array of composite fault types. Future research should aim to broaden the dataset to include a wider spectrum of disturbances and composite faults, thereby improving the model’s robustness and overall utility.

## Conclusion

This paper presents a MGCNN and SDTransformer model tailored for identifying power quality disturbances in microgrids, emphasizing their intricate temporal characteristics. The following conclusions are based on simulation experiments conducted in VSCode.

The proposed 1D-GAM enhances the detection capabilities for PQDs by integrating channel and sequence attention mechanisms. The MGCNN complements this by capturing both local details and global trends in PQDs through the use of variably sized convolutional kernels at different layers, improving sensitivity to minor signal variations and recognizing broader pattern changes for comprehensive temporal and spatial analysis. Building on these advancements, the MGCNN-SDTransformer model combines the SDTransformer’s multi-head attention mechanism to process signals concurrently across multiple subspaces, significantly improving accuracy in identifying and analyzing interdependencies among disturbances. Through various comparative experiments, the model’s optimal parameters were identified, enabling it to recognize 29 types of PQDs with a remarkable accuracy of 99.64% in noise-free environments and 98.85% at 20 dB SNR, showcasing strong noise immunity. Additionally, real-data experiments demonstrated its effectiveness in recognizing five types of disturbance signals, further validating the model’s robust generalization performance.

The dataset in this study lacks comprehensiveness; future work will include more complex grid data to enhance experiments and validate the model. Additionally, developing related hardware devices will help meet the evolving needs of new power systems.

## Supporting information

S1 Data (DOCX)
