# Supplementary material for: Classification of power quality disturbances in microgrids using a multi-level global convolutional neural network and SDTransformer approach
Source: PLoS One. 2025 Feb 12;20(2):e0317050. doi: 10.1371/journal.pone.0317050 (PMC11819554; doi:10.1371/journal.pone.0317050)
Supplement: S1 Data — (DOCX) [file pone.0317050.s001.docx]

**Table 4. 29 Categories of Recognition Accuracy**

| Labels | Disturbance type | Noise-free(%) | 50dB(%) | 30dB(%) | 20dB(%) |
| --- | --- | --- | --- | --- | --- |
| C0 | Nominal | 100 | 100 | 98.65 | 91.18 |
| C1 | Swell | 100 | 98.59 | 100 | 95 |
| C2 | Sag | 100 | 100 | 100 | 100 |
| C3 | Harmonics | 100 | 100 | 100 | 100 |
| C4 | Flicker | 100 | 95 | 97.75 | 98.67 |
| C5 | Interruption | 98.84 | 98.82 | 98.72 | 100 |
| C6 | Pulse | 100 | 100 | 100 | 98.89 |
| C7 | Oscillatory | 100 | 100 | 100 | 100 |
| C8 | gap | 100 | 97.78 | 100 | 100 |
| C9 | spike | 100 | 100 | 100 | 100 |
| C10 | Harmonics+Swell | 100 | 100 | 100 | 98.57 |
| C11 | Harmonics+Sag | 100 | 100 | 100 | 98.77 |
| C12 | Harmonics+Interruption | 100 | 100 | 98.89 | 98.65 |
| C13 | Harmonics+Flicker | 100 | 100 | 98.82 | 100 |
| C14 | Harmonics+Pulse | 100 | 100 | 100 | 100 |
| C15 | Harmonics+Oscillatory | 100 | 100 | 100 | 100 |
| C16 | Flicker+Swell | 100 | 100 | 100 | 100 |
| C17 | Flicker+Sag | 100 | 100 | 96.05 | 98.81 |
| C18 | Flicker+Oscillatory | 100 | 100 | 100 | 100 |
| C19 | Flicker+Pulse | 100 | 100 | 100 | 100 |
| C20 | Flicker+Interruption | 97.47 | 97.53 | 95.38 | 93.26 |
| C21 | Swell+Oscillatory | 100 | 100 | 100 | 100 |
| C22 | Sag+Oscillatory | 100 | 100 | 100 | 100 |
| C23 | Swell+Pulse | 100 | 100 | 100 | 100 |
| C24 | Sag+Pulse | 100 | 100 | 100 | 100 |
| C25 | Harmonics+Oscillatory+Swell | 98.67 | 100 | 100 | 98.61 |
| C26 | Harmonics+Oscillatory+Sag | 98.86 | 100 | 100 | 100 |
| C27 | Harmonics+Oscillatory+  Interruption | 95.71 | 96.2 | 98.73 | 97.3 |
| C28 | Harmonics+Oscillatory+Flicker | 100 | 100 | 100 | 100 |
| Total |  | 99.64 | 99.55 | 99.41 | 98.85 |

**Table 5. Comparison of model recognition accuracy**

| Different treatments | Noise-free（%） | 50dB（%） | 30dB（%） | 20dB（%） |
| --- | --- | --- | --- | --- |
| EMD | 98.64 | 98.41 | 98.21 | 97.88 |
| WPD | 99.83 | 98.61 | 98.74 | 98.7 |
| DWT | 99.65 | 99.35 | 99.2 | 98.71 |
| Raw Data | 99.64 | 99.55 | 99.41 | 98.85 |

**Table 6. Comparison of different levels of global convolution modules**

| Layers of Convolution Module | 30dB(%) | Training time (s) | number of parameters |
| --- | --- | --- | --- |
| MC=2 | 96.92 | 1139 | 27997 |
| MC=3 | 97.66 | 826 | 76669 |
| MC=4 | 98.18 | 806 | 235453 |
| MC=5 | 98.52 | 836 | 798781 |
| MGC=2 | 97.99 | 1212 | 33357 |
| MGC=3 | 99.01 | 987 | 98733 |
| MGC=4 | 99.41 | 1106 | 323693 |
| MGC=5 | 98.96 | 1401 | 1150445 |

**Table 7. Comparison of Different SDTransformer Network Depths**

| Base SDTransformer Layers | Noise-free(%) | 50db(%) | 30db(%) | 20db(%) | Training time (s) |
| --- | --- | --- | --- | --- | --- |
| 2 | 99.64 | 99.55 | 99.41 | 98.85 | 1106 |
| 4 | 99.39 | 99 | 98.87 | 97.87 | 1285 |
| 6 | 98.91 | 98.65 | 98.52 | 97.35 | 1394 |

**Table 8. Comparison of the number of MSA in SDTransformer network**

| Number of MSA | Noise-free(%) | 50db(%) | 30db(%) | 20db(%) | Training time (s) |
| --- | --- | --- | --- | --- | --- |
| 2 | 99.48 | 99.09 | 98.52 | 98.31 | 1105 |
| 4 | 99.64 | 99.55 | 99.41 | 98.85 | 1106 |
| 8 | 99.78 | 99.2 | 99.18 | 98.7 | 1149 |

**Table 9. Ablation experiments between different modules**

| Module | 30db Accuracy (%) |
| --- | --- |
| CNN | 97.15 |
| MGCNN | 97.65 |
| Transformer | 58.16 |
| SDTransformer | - |
| CNN-Transformer | 98.05 |
| CNN-SDTransformer | 98.18 |
| MGCNN-Transformer | 99.1 |
| MGCNN-SDTransformer | 99.41 |

**Fig 7. Recognition accuracy of 29 disturbance types under different networks**

**Fig 8. Disturbance integrated recognition rate under different networks**

| CNN-Bilstm | 98.14 |
| --- | --- |
| Transformer | 80.09 |
| 1D-CNN | 97.17 |
| LSTM | 80.2 |
| CNN-LSTM | 98.95 |
| MGC-Transformer | 99.55 |

**Table 10. Comparison of Recognition Rates of Different Models**

| Methodologies | Number of PQDs | 20dB | 30dB | Accuracy (%)  40dB | 50dB | Noise-free |
| --- | --- | --- | --- | --- | --- | --- |
| FDST+DT[27] | 13 | - | 97.49 | 98.8 | 99.28 |  |
| DWT+PNN[28] | 16 | 93.6 | 95.2 | 98.6 | - |  |
| CS+ DCNN[29] | 15 | - | 99.81 | 99.94 | - | 99.99 |
| GCNN+AFEN[30] | 12 | 96.62 | 98.98 | 99.26 | - |  |
| 1D VGG[31] | 17 | 96.74 | 98.49 | 98.93 | - |  |
| DAE[32] | 15 | 97.99 | 98.69 | 98.93 | - |  |
| MCF-TST[33] | 29 | 96.74 | 99.51 | - | 99.34 | 99.33 |
| MTF-EfficientNet[34] | 23 | 96.2 | 98.12 | 96.1 |  | 99.48 |
| DL-WMV[35] | 19 | - | 98.05 | 98.58 | 99.26 | - |
| MGCNN-SDTransformer | 29 | 98.85 | 99.41 | - | 99.55 | 99.64 |

**Table 11. Recognition results of real dataset**

| Disturbance type | Sample number | Accuracy(%) |
| --- | --- | --- |
| Nominal | 200 | 100 |
| Interruption | 200 | 99.5 |
| Pulse | 200 | 100 |
| 3rd Harmonics | 200 | 99.5 |
| 5th Harmonics | 200 | 99.5 |
| Average |  | 99.7 |
